# A case report of left circumflex stent infection and mycotic aneurysm: a rare but life-threatening complication of percutaneous coronary intervention

**DOI:** 10.1186/s43044-024-00442-0

**Published:** 2024-01-27

**Authors:** Swasthi S. Kumar, Sumanyu Suresh, Mohamed Iliyas, Jyothi Vijay, Vivek Pillai

**Affiliations:** 1 Department of Cardiology, Sree Chitra Tirunal Institute for Medical Sciences and Technology, Thiruvananthapuram, Kerala 695011 India; 2 Department of Cardiothoracic Surgery, Sree Chitra Tirunal Institute for Medical Sciences and Technology, Thiruvananthapuram, Kerala 695011 India

**Keywords:** Case report, Coronary artery aneurysm, Mycotic aneurysm, Percutaneous coronary intervention, Stent, Stent infection

## Abstract

**Background:**

Coronary stent infections are an uncommon but deadly complication of percutaneous coronary intervention. Mortality remains as high as 40–60% even with adequate treatment. We report such an interesting case of left circumflex stent (LCX) infection and mycotic aneurysm that was successfully managed with antibiotics and surgery.

**Case presentation:**

A middle-aged man who underwent percutaneous coronary intervention (PCI) to the left circumflex artery four weeks prior was referred as a case of pyrexia of unknown origin, not responding to antibiotics, and colchicine started for suspected Dressler syndrome. Although the inflammatory markers were elevated, the results of the blood culture did not show any growth. Echocardiography showed a doubtful echogenic structure in the left atrioventricular groove and mild pericardial effusion, and a stent infection was suspected. PET scan showed focal metabolic activity in the region of the LCX stent, with metabolically active supraclavicular and paratracheal lymph nodes, and a coronary angiogram revealed an aneurysm arising distal to the stented LCX. A diagnosis of stent infection and associated mycotic aneurysm was made, and the patient underwent surgery which included aneurysm repair, stent retrieval, and coronary artery bypass graft (CABG) to the major and terminal OM. The postoperative course was uneventful, and the patient was discharged without complications.

**Conclusions:**

It is important to investigate the possibility of coronary stent infection in individuals experiencing prolonged fever following PCI. PET scans and coronary angiograms can aid in diagnosis when echocardiograms are inconclusive. Adequate antibiotic therapy and timely surgery are crucial for successfully managing coronary stent infections.

## Background

Coronary stent infection is a rare but life-threatening complication following percutaneous coronary interventions. Despite adequate antibiotic therapy and surgical retrieval of infected stents, mortality remains as high as 40–60%. We report a case of coronary stent infection and mycotic aneurysm following LCX stenting that was successfully managed with surgery and antibiotics.

## Case presentation

**Fig. 1:**
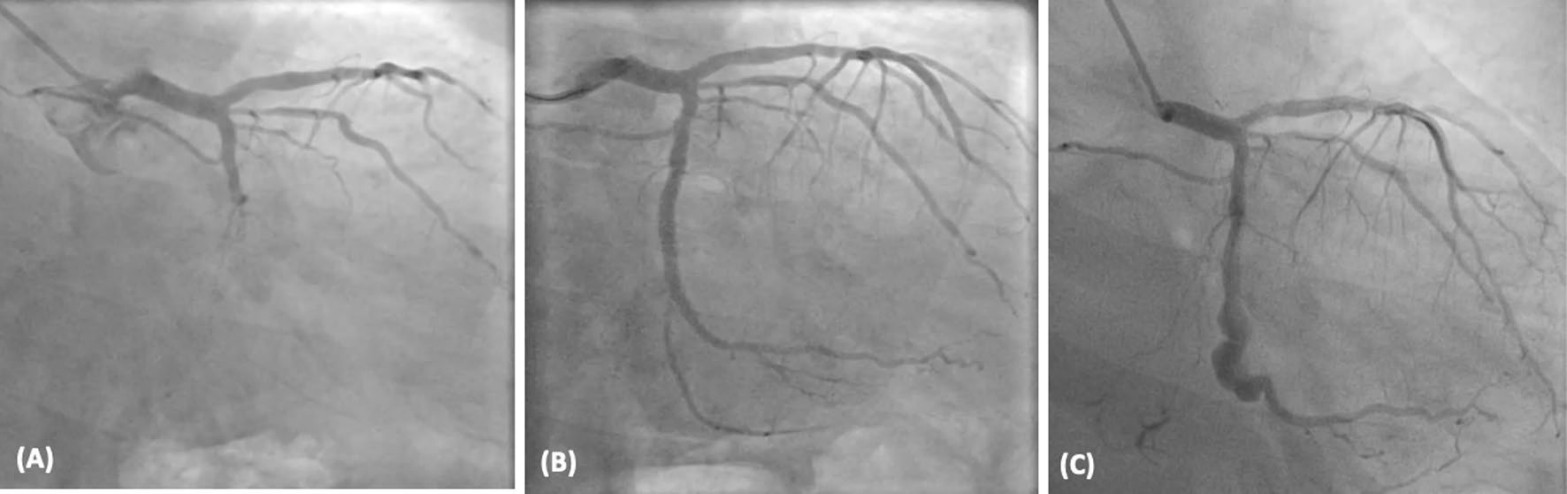
**A** Coronary angiogram done during first admission with chest pain showing total occlusion of LCX. **B** TIMI-III flow achieved after stenting. **C** Coronary angiogram taken to confirm the diagnosis of stent infection showing a mycotic aneurysm arising distal to the stented LCX

A middle-aged gentleman was referred to our centre with a fever of more than two weeks duration following a percutaneous coronary intervention (PCI) without any identifiable etiology. He had a history of anterior wall myocardial infarction ten years back, managed by PCI to the left anterior descending artery (LAD). He was recently admitted with new-onset angina at an outside hospital four weeks prior. Coronary angiogram (CAG) identified the left circumflex as the culprit vessel and showed a patent LAD stent. Subsequently, PCI to LCX was done with a drug-eluting stent, and the patient was discharged on dual antiplatelet therapy. One week post-discharge, the patient was re-admitted with high-grade fever with chills, which did not respond to the empirical antibiotics. With the background of recent myocardial infarction and PCI, Dressler syndrome was suspected. However, the patient failed to respond to colchicine. In addition, transoesophageal echocardiogram (TEE) showed an echogenic structure of 2.7 × 3.6cm in the left atrioventricular groove and mild pericardial effusion. After ruling out other foci of infection by preliminary investigations, the patient was referred to us for further evaluation with a high suspicion of stent infection (Fig. 1).

**Fig. 2:**
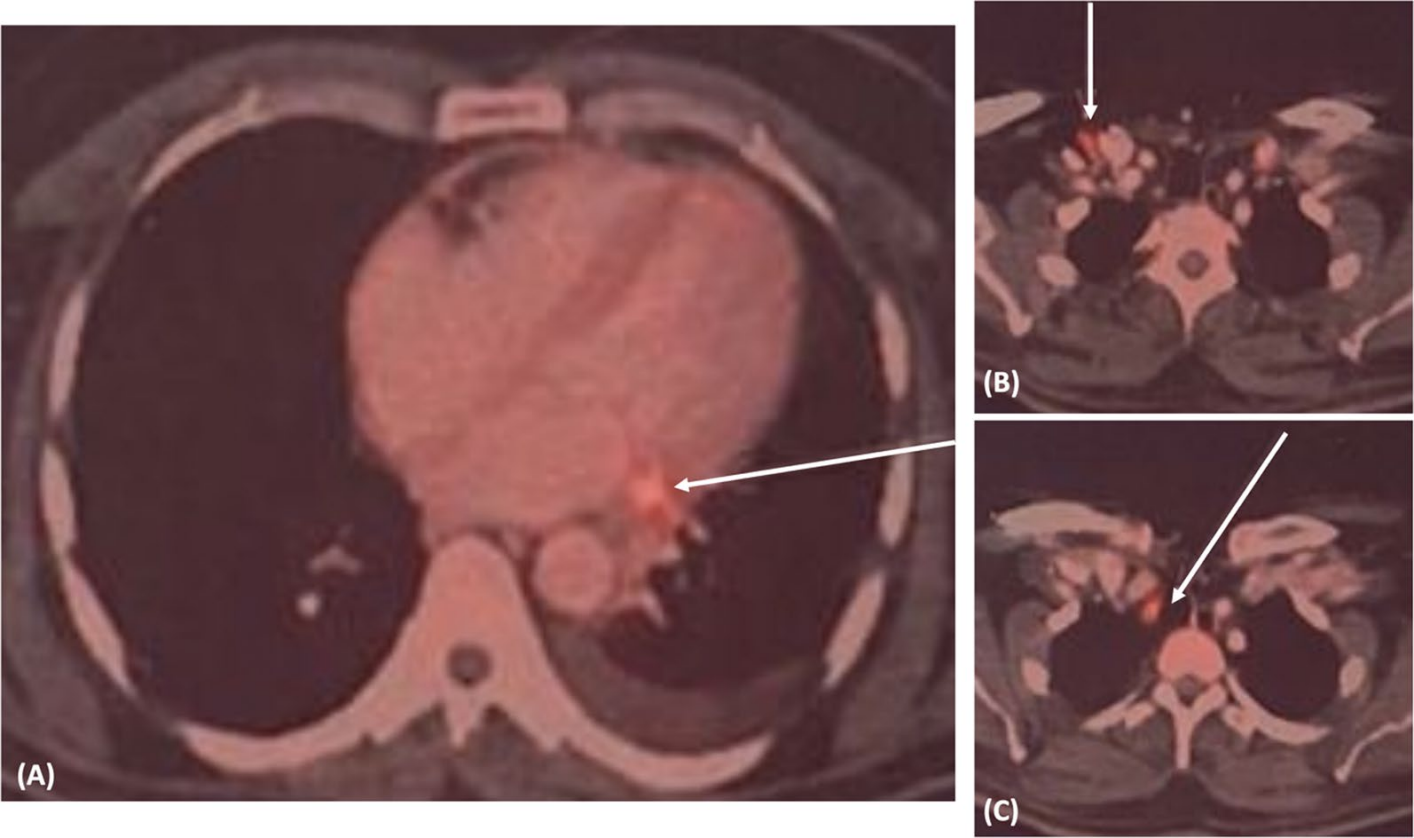
PET scan of the patient showing **A** focal metabolic activity along the stent in LCX, **B** metabolically active supraclavicular, and **C** right paratracheal lymph nodes

At presentation, the patient continued to have a high-grade fever; he was hemodynamically stable and cardiovascular examination was normal. ECG showed evidence of old AWMI. ECHO showed good left ventricular function with mild anterior wall hypokinesia and mild pericardial effusion. The hemogram showed mild neutrophilic leucocytosis and Inflammatory markers like ESR, CRP, and procalcitonin levels were elevated. Aerobic and anaerobic blood cultures revealed no growth. However, the patient was already on empirical antibiotics from the outside centre (Fig. 2).

**Fig. 3:**
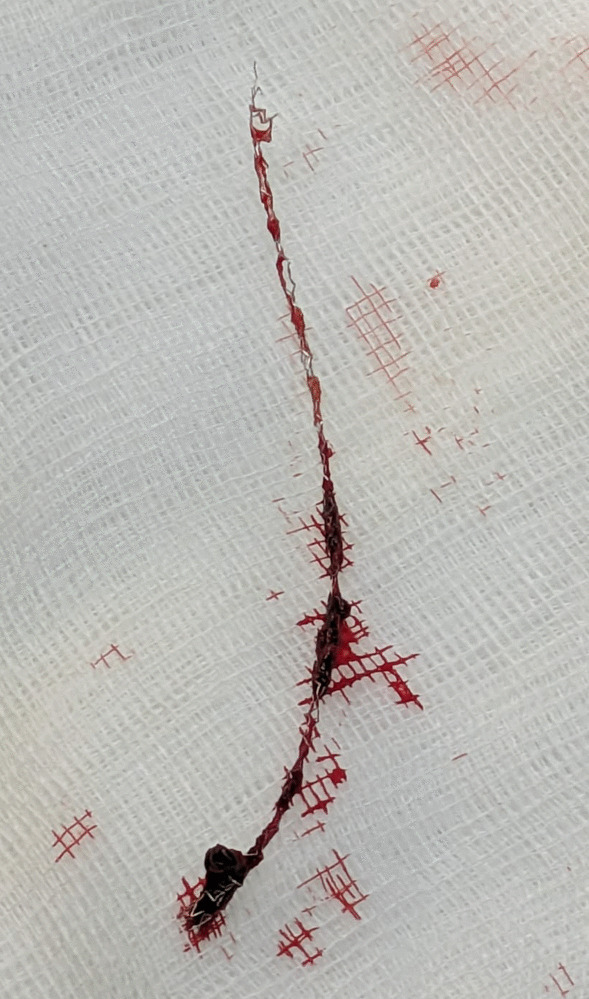
Surgical specimen of the retrieved stent and mycotic aneurysm segment of the left circumflex artery

CT angiogram was done to look for stent infection and showed a normally placed stent in proximal LCX. Mid and distal LCX could not be visualized due to motion artefacts. Mild pericardial effusion with subtle pericardial enhancement was noted, suggestive of pericarditis. PET scan showed focal metabolic activity in the region of the LCX stent, with metabolically active supraclavicular and paratracheal nodes, indicating a strong possibility of stent infection. CAG was done and showed an aneurysm arising distal to the stented LCX. A diagnosis of stent infection with mycotic aneurysm was confirmed, and the patient was advised to continue on antibiotic therapy. The inflammatory markers gradually normalized, and the patient underwent surgical repair. Dense pericardial adhesions were noted during surgery, and mid-LCX showed a 2 × 1cm aneurysm distal to the stent with a thrombus inside. The aneurysm was repaired after removing the thrombus and retrieving the LCX stent. Coronary artery bypass graft (CABG) was done with two saphenous vein grafts to major and terminal obtuse marginal (OM) arteries. The postoperative course was uneventful, and the patient fully recovered and was discharged without complications (Fig. 3).

## Discussion

Coronary stent infections are an infrequent and life-threatening complication of percutaneous coronary interventions. Mortality in cases of coronary stent infections has been reported as high as 40–60% [1]. With less than fifty reported cases worldwide, there is a need for more data on this topic. A possible diagnosis must be suspected in patients with coronary stent placement in the preceding four weeks or access site complications presenting with fever without apparent localization, leucocytosis, or bacteraemia. Demonstrating an infected coronary artery stent complex by autopsy or surgery gives a definitive diagnosis of coronary stent infection. Table 1 depicts the diagnostic criteria for coronary stent infection proposed by Dieter [2].

**Table 1:** Diagnostic criteria for coronary stent infections

Diagnostic criteria for coronary stent infections
**Definitive diagnosis:**
The demonstration of an infected coronary artery stent complex by autopsy or surgery
**Possible diagnosis:**
Any three of-
Placement of a coronary stent in the preceding three weeks
Access site complications or performing multiple procedures via the same arterial sheath
Bacteraemia
Fever without any obvious cause
Leucocytosis
Acute coronary syndrome
Imaging (Echo, CT, or MRI) suggestive of inflammation

Fever is the most common presentation. Stent infections may also present as recurrent stent thrombosis, acute myocardial infarction, and pericardial effusion [3]. In most cases, it occurs as an early presentation with onset between two days and four weeks [1]. However, very late presentations as long as five years post-stenting have been reported [4]. *Staphylococcus aureus* is the most common organism in 80% of cases, followed by *Pseudomonas aeruginosa* [1]. Risk factors for stent infection are the re-use of hardware like catheters, failure to maintain aseptic precautions during the procedure, local site infections and procedural complications like hematoma/ pseudo-aneurysms, repeated use of the same regional site for procedures, and prolonged placement of arterial sheaths [5]. Stent infections are more common with drug-eluting stents compared to bare metal stents. DES prevents the proliferation of neo-intima, and the uncovered stent struts serve as a nidus for bacterial infection [6].

First-line imaging modalities for diagnosis are echocardiography and coronary angiography. Echocardiography may sometimes pick up coronary aneurysms or collections surrounding infected stents. In our patient, TEE showed suspicion of an echogenic structure in the left AV groove, strengthening suspicion of stent infection. The presence of pericardial effusion may also be an indicator of stent infection. Cardiac CT, MRI, PET, and WBC scans may also help diagnose stent infections. A PET scan will show increased metabolic activity at the infected site, localizing the site of infection.

Mycotic coronary aneurysms and pseudoaneurysms, abscess formation, pericardial empyema, and purulent pericarditis are all dreaded complications [7]. Elison et al., 2012, reported a case of stent infection leading to abscess formation, which culminated in the death of the patient due to myocardial perforation and cardiac tamponade [8]. Coronary stent infections associated with infective endocarditis have also been reported [9]. Soman et al., 2015, reported a series of five cases of coronary stent infection caused by rapidly growing mycobacteria (RGM), which lead to infective endocarditis. Four out of five patients died due to complications of infective endocarditis, and the reuse of balloon angioplasty catheters was thought to be the plausible cause behind this complication [10]. Formation of coronary-cameral fistula secondary to coronary stent infection has also been reported [11].

Antibiotic therapy is the mainstay of treatment. Early-onset infections, arbitrarily defined as those with onset within ten days of stent placement, were likely to respond to antibiotic therapy alone [8]. Empirical antibiotic therapy should cover Staphylococcus aureus and Pseudomonas; prolonged antibiotic therapy lasting at least four weeks is recommended. However, foreign body infections may remain resistant to antibiotics until the source of infection has been removed from the body. Late-onset infections (with onset more than ten days after stent placement) and local complications require adequate antibiotic therapy along with surgical infected stent retrieval and aneurysm repair, removal of purulent pericardial fluid, granulomatous tissue, and revascularisation of the affected coronary vessel by CABG.

## Conclusions

It is important to investigate the possibility of coronary stent infection in individuals experiencing prolonged fever following PCI. PET scans and coronary angiograms can aid in diagnosis when echocardiograms are inconclusive. Adequate antibiotic therapy and timely surgery are crucial for successfully managing coronary stent infections.

## Data Availability

All details pertaining to the case are available from the corresponding author upon reasonable request.

## Abbreviations

CAG: Coronary angiogram
CABG: Coronary artery bypass graft
CRP: C-reactive protein
CT: Computed tomography
ECG: Electrocardiogram
ESR: Erythrocyte sedimentation rate
LAD: Left anterior descending
LCX: Left circumflex
MRI: Magnetic resonance imaging
OM: Obtuse marginal
PET: Positron emission tomography
PCI: Percutaneous coronary intervention
TEE: Transoesophageal echo
WBC: White blood cell
